# Post-Diagnostic Diet and Breast Cancer Outcomes: A Comprehensive Systematic Review of Prospective Cohort Studies and Randomized Clinical Trials

**DOI:** 10.3390/nu18162586

**Published:** 2026-08-07

**Authors:** Loubna Enezari, Matteo Della Porta, Serena Mazzucchelli, Fabio Corsi, Jeanette A. Maier, Roberta Cazzola

**Affiliations:** Department of Biomedical and Clinical Sciences, University of Milano, via G. B. Grassi, 74, 20174 Milan, Italy; nizariloubna@outlook.fr (L.E.); serena.mazzucchelli@unimi.it (S.M.); fabio.corsi@unimi.it (F.C.); jeanette.maier@unimi.it (J.A.M.)

**Keywords:** breast cancer survivorship, post-diagnostic diet, dietary patterns, diet quality, all-cause mortality, nutritional assessment methods, malnutrition, food frequency questionnaire

## Abstract

**Background/Objectives:** Diet is increasingly recognized as a modifiable factor in cancer survivorship; however, the impact of post-diagnostic dietary habits on breast cancer (BC) outcomes remains uncertain. This systematic review aimed to evaluate the association between diet after BC diagnosis and clinical outcomes and to provide an updated synthesis of the evidence, with particular emphasis on dietary assessment methods, methodological heterogeneity, and evidence gaps. **Methods:** We systematically reviewed randomized controlled trials (RCTs) and prospective cohort studies that investigated post-diagnostic dietary exposures among women with BC. Outcomes of interest included all-cause mortality (ACM), breast cancer-specific mortality (CSM), recurrence (R), overall survival (OS), and disease-free survival (DFS). Sources of methodological heterogeneity were assessed, and findings were synthesized using a structured narrative approach. **Results:** A total of 39 studies were included (8 RCTs and 31 prospective cohort studies), comprising over 90,000 participants. Evidence from RCTs, including low-fat and multi-component interventions, showed largely null or inconsistent effects on BC-specific outcomes. Findings from cohort studies were heterogeneous. Associations for individual nutrients and food groups were generally inconsistent across studies, whereas healthier dietary patterns and higher diet quality scores were associated with reduced ACM. Associations with BC-specific outcomes were less consistent. Overall, the available evidence was characterized by substantial methodological heterogeneity, overlapping study populations, and low-to-moderate certainty. **Conclusions:** Current evidence suggests that post-diagnostic dietary habits may contribute more to overall survivorship than to BC-specific prognosis. Methodological heterogeneity, particularly in dietary assessment, should be taken into account when interpreting findings. The principal contribution of this review is the identification of important methodological limitations and evidence gaps that currently limit confidence in the available literature. Future research should employ more accurate dietary assessment methods and comprehensive nutritional evaluation to clarify the role of nutrition in BC outcomes.

## 1. Introduction

Breast cancer (BC) is the most common cancer among women worldwide, with more than 2.3 million new cases estimated annually. Although the incidence of BC continues to rise, mortality has steadily declined over recent decades, largely due to the expansion of screening programs and advances in diagnostic and therapeutic strategies [1,2,3]. Several factors contribute to cancer development, including dietary and lifestyle habits, radiation exposure, and hormonal factors [4]. Research indicates that an estimated 4.2–5.2% of all cancer cases each year can be directly linked to inadequate nutrition, underscoring the role of dietary factors as significant modifiable elements in the etiology and prevention of cancer [5].

Beyond its role in cancer development, dietary habits and nutrition are increasingly recognized as an important component of the cancer care pathway, both during treatment and throughout survivorship [6,7]. Adequate nutritional intake may improve treatment tolerance, reduce therapy-related side effects, support immune function, and enhance recovery. Furthermore, it has been suggested that dietary habits after diagnosis may influence long-term outcomes, such as all-cause mortality, recurrence, and quality of life. However, while the role of diet in cancer prevention is well established [8], there is less consensus regarding the impact of post-diagnostic nutrition on cancer prognosis, particularly in oncology patients.

In recent decades, a growing body of research has investigated the associations between post-diagnostic dietary patterns, specific foods, and prognostic outcomes in patients with cancer [9]. Despite this increasing interest, findings remain heterogeneous and often inconclusive, highlighting the need for more robust and methodologically consistent research. In particular, variability in dietary assessment methods, exposure definitions, and outcome measures may contribute to the heterogeneity observed across studies. A major challenge in this field lies in the accurate assessment of dietary intake. Common methods include 24 h dietary recalls, food frequency questionnaires (FFQs), and food diaries (FDs) [10], each with specific advantages and limitations.

The 24 h recall is widely used but captures only short-term intake and may require repeated administration to estimate habitual diet, increasing participant burden and study costs [11]. FFQs are commonly employed in large epidemiological studies due to their practicality and low cost; however, they are prone to measurement error and often overestimate food and nutrient intake compared to more precise methods [11,12]. In contrast, FDs prospectively record food intake and may provide more precise estimates of short-term dietary intake, with reduced recall bias [11]. However, they are also subject to important limitations, including participant burden, potential reactivity, and a limited ability to capture long-term habitual dietary patterns. Differences in dietary assessment methods can significantly influence observed associations. For example, Bingham et al. reported that fat intake assessed using FDs was associated with increased BC risk, whereas no significant association was found when intake was assessed using FFQs [13]. However, while these observations suggest that methodological differences in dietary assessment may influence study findings, no direct comparison of results according to assessment method was performed in most studies, and such differences should therefore be interpreted as potential contributors rather than definitive explanations of inconsistent results.

In addition to measurement challenges, cancer patients often experience disease- and treatment-related factors that directly affect dietary intake. Patients with BC may experience changes in taste, smell, and appetite, leading to altered food preferences and reduced intake. Common symptoms, including early satiety, nausea, vomiting, and gastrointestinal dysfunction, can further impair nutritional status. Psychological factors such as anxiety and depression, as well as cancer-related pain, may exacerbate these difficulties [14]. As a result, oncology patients are at high risk of malnutrition.

In BC patients, failure to identify and manage malnutrition is associated with adverse clinical outcomes, poorer prognosis, and increased mortality [15]. Importantly, malnutrition can also occur in individuals with obesity due to inadequate nutrient intake. Although it is largely preventable and potentially reversible with appropriate nutritional interventions, undernutrition often remains underdiagnosed in clinical practice due to limited awareness and a lack of standardized screening protocols. Malnutrition is a clinically relevant factor in cancer patients; however, it was rarely assessed in the studies included in this review and was often insufficiently captured. Therefore, it should be considered primarily as a potential source of residual confounding and a limitation of the available evidence rather than a directly evaluated exposure. The lack of well-defined, evidence-based dietary recommendations for cancer patients generates considerable uncertainty and underscores the necessity for research that comprehensively integrates dietary intake with indicators of nutritional status. This gap in information may expose patients to inaccurate or misleading guidance, particularly from non-scientific sources.

Given these considerations, this systematic review aims to evaluate the evidence from randomized controlled trials (RCTs) and prospective cohort studies investigating the association between post-diagnostic dietary habits and BC outcomes. Attention is given to methodological aspects of dietary assessment and to factors related to nutritional status, including the potential role of malnutrition, which represent key distinguishing features of this review compared with previous literature. Compared with previous reviews, this work provides an updated and methodologically focused synthesis of the available evidence, integrating recent studies with a specific emphasis on prospective study designs, heterogeneity in dietary assessment methods, and variability in exposure and outcome definitions.

Importantly, the primary objective of this review is not to derive a pooled quantitative estimate of the effect of post-diagnostic dietary exposures on breast cancer outcomes, but rather to critically evaluate the quality, consistency, methodological robustness, and clinical interpretability of the available evidence. By identifying major methodological limitations, evidence gaps, and areas requiring further investigation, this review aims to provide a comprehensive assessment of the current state of knowledge and inform priorities for future research.

## 2. Materials and Methods

### 2.1. Search Strategy

A systematic literature search was conducted in PubMed/MEDLINE, CENTRAL, and Scopus in accordance with the PRISMA 2020 guidelines [16]. The PRISMA 2020 checklist is provided in the Supplementary Materials. The initial search was performed on 27 May 2025. To ensure completeness and currency of the evidence base, the search was updated on 5 May 2026, using the same databases, Boolean search strategy, and eligibility criteria. The updated search was conducted before manuscript finalization to ensure inclusion of the most recent eligible studies. No additional eligible studies were identified.

The selection of databases (PubMed/MEDLINE, CENTRAL, and Scopus) was guided by the aim of ensuring comprehensive coverage of biomedical, clinical, and interdisciplinary literature relevant to the research question. PubMed/MEDLINE was selected as the primary biomedical database, CENTRAL to optimize retrieval of randomized controlled trials, and Scopus to extend coverage to multidisciplinary domains, including nutrition, public health, and behavioral sciences. This approach is consistent with the PRISMA 2020 recommendations, which do not prescribe a fixed set of databases but emphasize transparent reporting and justification of information sources (Page et al., 2021 [16]). The combination of these databases was considered appropriate to capture the core body of evidence while ensuring feasibility and reproducibility.

The search strategy was developed a priori and combined controlled vocabulary and free-text terms related to diet and BC outcomes. The search strategy combined free-text terms with database-specific indexing features (e.g., PubMed automatic term mapping), which allows the identification of records indexed with controlled vocabulary such as MeSH terms. The use of free-text terms was intended to maximize sensitivity by capturing recently indexed articles and heterogeneous terminology commonly used in nutritional and epidemiological research. The search strategy was developed to balance sensitivity and specificity, prioritizing key exposure terms (foods, nutrients, and dietary patterns) to limit retrieval of non-relevant literature (e.g., general lifestyle interventions or non-dietary exposures), a recognized challenge in nutritional oncology.

The main search components included the following:

- Dietary exposure: diet, nutrition, dietary pattern, food, nutrients;
- Disease: breast cancer, breast tumor;
- Population: patient;
- Outcomes: survival, mortality, recurrence.

Terms related to parenteral or enteral nutrition, vitamins, minerals, and supplements were excluded from this analysis. Alcohol consumption was not included as a separate exposure term, as the focus was on overall dietary patterns and food-based exposures. No restrictions were applied at the search stage, other than language (English) and human studies. Eligibility criteria related to study design (prospective cohort studies and randomized controlled trials) were applied during the screening phase to ensure inclusion of studies with an appropriate temporal structure for assessing post-diagnostic exposures and outcomes. The focus on prospective and randomized study designs was intended to improve internal validity by reducing recall bias and ensuring temporal alignment between dietary exposure and clinical outcomes.

In accordance with PRISMA-S recommendations, full search strategies for all databases are provided in the Supplementary Materials to ensure transparency and reproducibility (Supplementary Tables S1–S3).

### 2.2. Eligibility Criteria

Studies were selected according to the PICOS framework, as summarized in Table 1.

Inclusion criteria were:

- Population: adult women (≥18 years) with a diagnosis of BC.
- Exposure/Intervention: assessment of dietary habits, dietary patterns, or diet quality after BC diagnosis, including studies with repeated dietary assessments or analyses incorporating both pre- and post-diagnostic dietary data relevant to prognosis.
- Outcomes: overall survival (OS), all-cause mortality (ACM), breast cancer-specific mortality (CSM), disease-free survival (DFS), cancer progression (CP), or recurrence.
- Study design: prospective cohort studies and RCTs.
- Dietary assessment tools: FFQs, FDs, or 24 h dietary recalls.

Exclusion criteria included non-human studies, reviews, case reports, conference abstracts, studies evaluating non-dietary interventions, and investigations that incorporated other treatments alongside dietary interventions, such as physical exercise or alternative therapies. Additionally, studies that did not report on breast cancer-related outcomes were also excluded.

Outcome definitions were based on those reported in the original studies and may have varied across studies, particularly for disease-free survival and recurrence. In general, all-cause mortality (ACM) was defined as death from any cause, breast cancer-specific mortality (CSM) as death attributable to BC, recurrence as the return of BC after initial treatment, disease-free survival (DFS) as the time from diagnosis or treatment to recurrence or death, and overall survival (OS) as the time from diagnosis to death from any cause.

For this review, “breast cancer survivors” were defined as women from the time of diagnosis onwards, regardless of treatment phase, provided that dietary exposure was assessed after diagnosis, or—if assessed before diagnosis—was considered representative of habitual dietary patterns and analyzed in relation to post-diagnostic outcomes. This approach applies to certain trial-based analyses (e.g., WHI), in which dietary exposure preceded diagnosis but was evaluated in relation to post-diagnostic outcomes.

Alcohol intake was not considered as a standalone exposure in this review; however, it was included when incorporated within composite dietary patterns, diet quality indices, or multi-component dietary interventions.

### 2.3. Study Selection and Data Extraction

Two reviewers (L.E. and M.D.P.) independently screened titles and abstracts, followed by full-text assessment of potentially eligible studies, using the Rayyan web application. Disagreements were resolved by discussion with a third reviewer (R.C.).

A manual search was conducted by screening the reference lists of included studies and relevant reviews, identifying 22 additional records.

Data was extracted using standardized forms and included study design, population characteristics, dietary exposure, outcome measures, follow-up duration, and adjustment for potential confounders. When available, information on multivariable adjustments was also recorded. Across studies, statistical models generally accounted for key clinical and lifestyle factors, including age, tumor characteristics, treatment, body mass index, and behavioral variables such as smoking and physical activity.

### 2.4. Risk of Bias Assessment

Risk of bias was assessed independently by two reviewers (L.E. and M.D.P.) using validated tools appropriate to the study design:

- the Cochrane Risk of Bias tool (RoB 2) for RCTs [17];
- the Newcastle–Ottawa Scale (NOS) for prospective cohort studies [18].

Discrepancies were resolved by discussion with a third reviewer (R.C.). Results of risk-of-bias assessments are detailed in Supplementary Tables S4 and S5. In brief, the main sources of bias in RCTs included lack of blinding, deviations from intended interventions, and incomplete outcome data, while cohort studies were primarily affected by residual confounding, exposure misclassification, and incomplete adjustment for clinical and lifestyle variables.

### 2.5. Data Synthesis

Due to substantial heterogeneity in dietary exposures (e.g., foods, nutrients, and dietary patterns), assessment methods (FFQs, dietary records), outcome definitions, and reporting (e.g., categorical vs continuous measures), a quantitative meta-analysis was not considered appropriate. This heterogeneity was systematically evaluated across predefined domains, including dietary exposure definition, assessment methods, timing of exposure measurement, clinical characteristics, and outcome reporting. In addition, multiple publications derived from the same large cohorts (e.g., WHI, NHS/NHSII, WHEL, LACE) contributed overlapping and potentially non-independent data, further limiting the feasibility of deriving independent effect estimates suitable for pooling.

Specifically, several sources of heterogeneity were identified: (i) Dietary assessment methods, which varied across studies (e.g., FFQs with different structures, 24 h recalls, and dietary records) and differed in validity and ability to capture habitual intake; (ii) Timing of dietary exposure assessment, including pre-diagnostic, post-diagnostic, and mixed or repeated measures, limiting comparability of exposure definitions; (iii) Dietary exposure definitions, encompassing individual nutrients, foods, dietary patterns, and multiple diet quality indices, often based on non-comparable scoring systems; (iv) Exposure categorization and statistical reporting, with effect estimates expressed using tertiles, quartiles, quintiles, or continuous measures; (v) Clinical heterogeneity, including variability in cancer stage, tumor receptor status (ER, PR, HER2), and treatment modalities (e.g., chemotherapy, radiotherapy, hormone therapy), which may influence prognosis and modify diet–outcome associations; (vi) Follow-up duration, which ranged widely across studies and affected comparability of outcome ascertainment; and (vii) Outcome definitions, particularly for recurrence and disease-free survival, were not standardized and were defined inconsistently across studies. This variability in outcome definitions represents a relevant source of heterogeneity and should be considered when interpreting the findings across studies.

Taken together, this multidimensional heterogeneity affected both the clinical comparability and statistical combinability of the data and precluded meaningful quantitative synthesis, including specific exposures or outcomes. Furthermore, the objective of the present review was not limited to estimating a summary measure of effect, but to evaluate the overall strength, consistency, and methodological quality of the available evidence. Given the substantial heterogeneity and limitations of the literature, a structured narrative synthesis was considered more consistent with the review objective and more informative for identifying evidence gaps and research priorities than the generation of pooled estimates.

Therefore, findings from randomized controlled trials originating from the same trial populations (e.g., WINS and WHI) were interpreted at the trial level rather than as independent pieces of evidence.

A structured narrative synthesis was therefore conducted, organized by dietary exposure and outcome category. This approach is consistent with guidance from the Cochrane Handbook for Systematic Reviews of Interventions [19], which recognizes that quantitative pooling may not be appropriate when substantial clinical and methodological heterogeneity limits meaningful comparability across studies, and with the SWiM (Synthesis Without Meta-analysis) reporting guideline for systematic reviews in which a structured narrative synthesis is considered more appropriate than statistical pooling [20].

The review protocol was prospectively registered in PROSPERO, registration No. CRD42024539276.

## 3. Results

### 3.1. Study Selection and Study Characteristics

**Figure 1:**
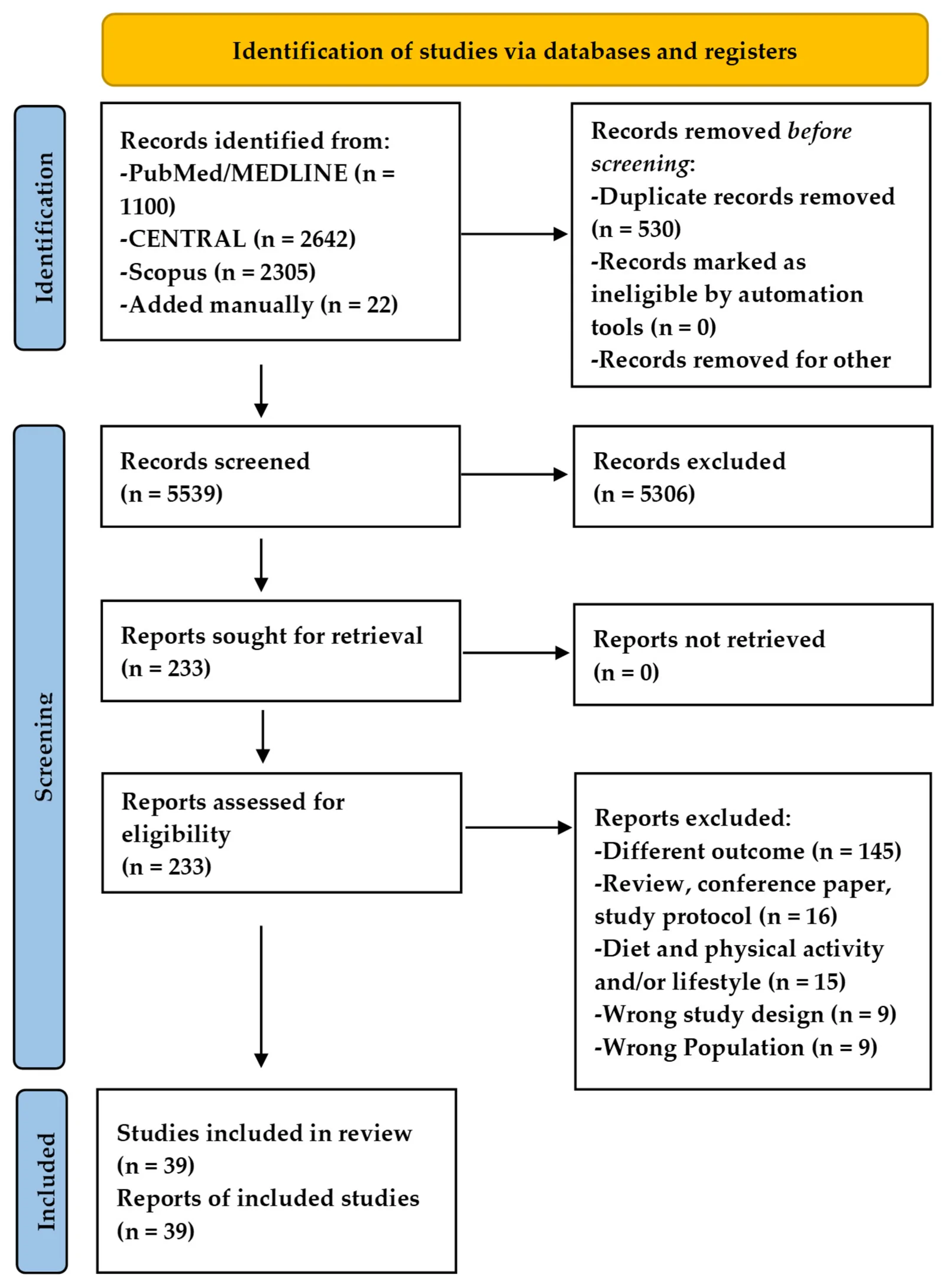
PRISMA flow diagram.

The systematic literature search identified a total of 6069 articles from PubMed/MEDLINE, CENTRAL, and Scopus, including 1100, 2642, and 2305 records, respectively. In addition, 22 articles were identified through a manual search conducted by the authors. After the removal of 530 duplicate records, 5539 articles were screened based on title and abstract. Of these, 5306 records were excluded. The remaining 233 articles underwent full-text screening, which resulted in the exclusion of 194 articles for the following reasons: different outcomes (*n* = 145), review articles, conference papers, or study protocols (*n* = 16), combined evaluation of diet and physical activity (*n* = 15), inappropriate study design (*n* = 9), and inclusion of an ineligible population (*n* = 9). Ultimately, 39 studies met the eligibility criteria and were included in the systematic review. The PRISMA flow diagram is shown in Figure 1.

Outcome measures were not fully standardized across studies, particularly for recurrence and disease-free survival, which may have been defined differently depending on the study design. This variability represents a relevant source of heterogeneity and should be considered when interpreting findings across studies.

Among the included studies, eight were RCTs evaluating the effects of dietary interventions on BC outcomes, recurrence, DFS, OS, breast cancer–specific mortality (CSM), and ACM. Details of the RCTs are reported in Table 2. In addition, 31 prospective cohort studies investigated the associations between post-diagnosis nutrition and BC outcomes and/or ACM; their main characteristics and findings are summarized in Table 3 and Table 4.

The number of participants across all included studies ranged from 114 to 13,270 participants included in the 39 publications considered in this systematic review. The total number of participants across studies was approximately 92,713; however, this figure reflects the sum of study-specific populations and may include overlapping cohorts across multiple publications.

The quality assessment of the studies included in the review revealed that RCTs were primarily rated as having a high risk of bias, with seven studies falling into this category. Only one RCT was classified as having a moderate risk of bias. In contrast, the prospective cohort studies showed a better overall quality. A total of 22 studies were categorized as having moderate quality, five studies were classified as lower quality, and four studies were deemed to possess higher quality (Supplementary Tables S4 and S5). The main sources of bias identified in RCTs included lack of blinding, deviations from intended interventions, and incomplete outcome data. In contrast, cohort studies were primarily affected by residual confounding, exposure misclassification, and incomplete adjustment for key clinical and lifestyle variables.

### 3.2. Randomized Controlled Trials

Eight RCTs met the inclusion criteria and evaluated the effects of nutritional interventions on BC outcomes, including recurrence, DFS, OS, CSM, and ACM. Detailed characteristics of the RCTs are reported in Table 2.

**Table 2:** Characteristics and main results of RCTs.

First Author, Year	Study (Country)	Tumor Receptor Status	Therapy	Years of Diagnosis	Cases Analyzed	Cancer Stage	Follow-Up (Median, Years)	Dietary Interventions	Outcomes	Main Findings
Reddy, 2005 [21]	WINS (USA)	ER	HT CT	1994–2001	2437 IG = 975 CG = 1462	NR	5	LFD	R DFS OS	LFD decreased R and increased DFS
Chlebowski, 2006 [22]	WINS (USA)	ER PR	HT CT	1994–2001	2437 IG = 975 CG = 1462	I II A II B III	5	LFD	R DFS OS	No difference between IG and CG
Chlebowski, 2017 [23]	WHI RCT (USA)	ER PR HER2	NR	1993–1998	3030 IG = 1176 CG = 1854	I II III	8.5	LFD	CSM ACM	LFD decreased ACM
Chlebowski, 2018 [24]	WHI RCT (USA)	ER PR HER2	CT RT HT	1993–1998	1764 IG = 671 CG = 1093	NR	11.5	LFD	OS	LFD increased OS
Chlebowski, 2020 [25]	WHI RCT (USA)	ER PR HER2	CT RT HT	1993–1998	3374 IG = 1299 CG = 2075	I II III	19.6	LFD	ACM CSM	LFD decreased ACM
Zheng, 2018 [26]	WHI RCT (USA)	ER PR	NR	1993–1998	1182 IG = 448 CG = 734	I II III Unk	13.3	Energy-adjusted DII	ACM CSM	No difference between IG and CG
Pierce, 2007 [27]	WHEL (USA)	ER PR	RT CT HT	1991–2000	3088 IG = 1537 CG = 1551	I II III A	7.3	Healthy Diet (LFD+ high in fruits, vegetables, and fiber)	ACM DFS	No difference between IG and CG
Berrino, 2024 [28]	Diana 5 Italy	ER PR HER2	NR	2008–2012	1542 IG = 769 CG = 773	NR	5	Mediterranean-macrobiotic dietary intervention	R	No difference between IG and CG

Abbreviations: ACM, all-cause mortality; CG, control group; CSM, breast cancer-specific mortality; CT, chemotherapy; DFS, disease-free survival; DII, dietary inflammatory index; ER, estrogen receptor; HER2, human epidermal growth factor receptor 2; HT, hormone therapy; IG, intervention group; LFD, low-fat diet; NR, not reported; OS, overall survival; PR, progesterone receptor; R, recurrence; RT, radiotherapy; Unk, unknown; WHEL, Women’s Healthy Eating and Living Study; WHI, Women’s Health Initiative; WINS, Women’s Intervention Nutrition Study. The included RCTs examined different molecular subtypes of BC, including estrogen receptor–positive disease (ER) [21], combined estrogen receptor and progesterone receptor positivity (ER/PR) [22,26,27,29], and combined ER, PR, and HER2 expression (ER/PR/HER2) [23,24,25,28]. Participants received different medical therapies, including hormone therapy (HT) and chemotherapy (CT) [21,22], or combinations of CT, radiotherapy (RT), and HT [24,25,27]. In three studies, the specific therapy administered was not reported [23,26,28].

Most RCTs enrolled women diagnosed with stage I to III BC [22,23,25,27], while one study also included participants with an unspecified stage beyond I–III [26]. In three studies, cancer stage at diagnosis was not reported [21,24,28]. Median follow-up duration ranged from 5 to 19.6 years.

Seven RCTs were conducted in the United States, and one was conducted in Italy (Table 2). The majority of trials primarily examined the effects of a low-fat dietary pattern (LFD) on ACM and/or BC-related outcomes, whereas DIANA-5 evaluated a multi-component dietary intervention based on Mediterranean and macrobiotic principles. The intervention encouraged increased consumption of whole grains, legumes, soy products, vegetables, fruits, nuts, olive oil, and fish, and avoidance of refined foods, sugars, red and processed meat, dairy products, and alcohol, reflecting a multi-component dietary intervention rather than a single-nutrient modification.

Dietary intake was assessed using FFQs in all major trials.

The Women’s Intervention Nutrition Study (WINS), the Women’s Health Initiative (WHI) dietary modification trial, and the Women’s Healthy Eating and Living (WHEL) Study represent the three largest and longest RCTs conducted in the United States. All trials compared intensive dietary intervention with a control condition characterized by minimal nutritional counseling and no structured dietary modification.

The WINS trial randomized 2437 women aged 48–79 years with stage I or II BC to either a dietary intervention group (IG; *n* = 975) or a control group (CG; *n* = 1462). After a median follow-up of 5 years, women in the IG achieved a significantly lower dietary fat intake and greater weight loss compared with those in the CG [21] (Table 2). Reddy et al. reported a 24% reduction in the risk of BC recurrence in the IG, although this effect was attenuated during the post-intervention follow-up period [21,30]. DFS was higher among women in the IG, whereas no statistically significant differences in OS were observed between groups [17]. An interim analysis conducted by Chlebowski et al. after a median follow-up of 60 months similarly showed no significant difference in OS between the two groups [22] (Table 2).

The WHI dietary modification trial represents the largest women’s health prevention study conducted to date. Enrolment occurred between 1993 and 1998, and the trial concluded in 2005. After a median dietary intervention duration of 8.5 years, Chlebowski et al. evaluated the long-term effects of the WHI dietary intervention in three separate publications [23,24,25]. Overall, the results indicated reductions in ACM without consistent effects on BC-specific outcomes (Table 2). In analyses published in 2017 and 2020, LFD was significantly associated with reduced ACM [23,25], while the 2018 analysis reported a significant association with improved OS [24].

It should be noted that several publications from the WINS and WHI trials represent multiple analyses of the same underlying study populations and were therefore considered collectively rather than as independent sources of evidence.

Zheng et al. evaluated the association between the energy-adjusted Dietary Inflammatory Index (e-DII) and mortality outcomes within the WHI cohort [26]. No overall association was observed between e-DII and either ACM or CSM. However, stratified analyses demonstrated an inverse association between e-DII and ACM among women with ER-positive BC [26] (Table 2).

The WHEL Study was designed to evaluate the effectiveness of a low-fat, high-vegetable diet in increasing circulating carotenoid concentrations and reducing additional BC events and early mortality among women diagnosed with early-stage invasive BC within four years of diagnosis. A total of 3088 participants were randomized to an IG (*n* = 1537) or a CG (*n* = 1551). After a median follow-up of 7.3 years, no statistically significant differences were observed between the intervention and control groups with respect to additional BC events or mortality outcomes [27] (Table 2).

The DIANA-5 trial enrolled 1542 women diagnosed with BC to evaluate the effectiveness of a dietary intervention based on Mediterranean and macrobiotic dietary principles (macro-Mediterranean diet) in reducing recurrence. The intervention encouraged increased consumption of whole grains, legumes, soy products, vegetables, fruits, nuts, olive oil, and fish, and avoidance of refined foods, potatoes, sugars, desserts, red and processed meat, dairy products, and alcohol, reflecting a multi-component dietary intervention rather than a single-nutrient modification. Both the intervention and control groups received the 2007 American Institute for Cancer Research/World Cancer Research Fund recommendations for cancer prevention. After a median follow-up of 5 years, no reduction in BC recurrence was observed in the intervention group compared with controls [28] (Table 2).

Overall, RCTs yielded heterogeneous and often inconsistent results, with limited evidence of an effect on BC–specific outcomes, whereas reductions in ACM were more frequently observed.

### 3.3. Prospective Cohort Studies

Thirty-one prospective cohort studies reporting the results of analyses based on FFQs and/or 24 h dietary recalls were identified, involving women drawn from thirteen distinct cohorts. The timing of dietary assessments exhibited considerable variability across different studies. Some cohorts conducted dietary evaluations after breast cancer diagnosis, while others utilized pre-diagnostic or baseline dietary data, often incorporating repeated measures that spanned both pre- and post-diagnostic periods. This divergence in exposure timing represents a significant methodological consideration, as pre-diagnostic assessments may capture long-term habitual dietary patterns rather than reflecting dietary alterations following diagnosis. Consequently, this variability in exposure timing should be regarded as a critical methodological limitation when comparing findings across studies. Overall, findings from prospective cohort studies were heterogeneous, particularly for breast cancer–specific outcomes.

Specifically, the cohorts comprised the Health, Eating, Activity, and Lifestyle (HEAL) Study [31], the Diet, Cancer, and Health (DCH) cohort [32], the Collaborative Women’s Longevity Study (CWLS) [33], the European Prospective Investigation into Cancer and Nutrition (EPIC) cohort [34,35], the Pathways Study [36], Cancer Prevention Study II (CPS-II) [37], the Nurses’ Health Study (NHS) [38,39,40,41,42,43,44,45,46,47], the Breast Cancer Family Registry (BCFR) [48], the Third National Health and Nutrition Examination Survey (NHANES III) [49], the Life After Cancer Epidemiology (LACE) Study [50,51], the Shanghai Breast Cancer Survival Study (SBCSS) [29,52,53], the Women’s Healthy Eating and Living (WHEL) Study [54,55], and the Women’s Health Initiative (WHI) [56,57]. In addition, three studies utilized cohorts that were not explicitly named [58,59,60].

**Table 3:** Nutritional characteristics of Cohort studies.

Cohort Name	Tools	Parameters Evaluated	Data Collection	Timing of Dietary Assessment (Relative to Diagnosis)	Reference
HEAL	122-items WHI FFQ	Energy, fiber, carbohydrates	24 months after BC diagnosis	Post-diagnostic	[31]
DCH	192-items FFQ	All food groups	At enrollment and five years after	Mixed (pre- and post-diagnostic)	[32]
CWLS	126-items FFQ	Macronutrient and micronutrient	Within 5 years of BC diagnosis	Post-diagnostic	[33]
EPIC	260-items FFQ Semi-quantitative FFQ 7-day dietary records	All food groups Meals	At enrollment	Pre-diagnostic	[34,35,61]
Pathway study	139-items FFQ 3-day dietary records	All food groups Dietary pattern	2 weeks after BC diagnosis	Post-diagnostic	[36]
CPS-II	60-items FFQ	All food groups	At enrollment and every 2 years	Mixed	[37]
NHS, NHSII	61-items FFQ 130-items FFQ	All food groups	At enrollment and every 4 years	Mixed	[38,39,40,41,42,44,45,46,47]
BCFR	108-items FFQ	All food groups	One year before BC diagnosis	Pre-diagnostic	[48,62]
NHANES III	24 h dietary recall	Food and beverages consumed in the past 24 h	At enrollment	Pre-diagnostic	[49]
LACE	122-items FFQ	All food groups	At enrollment and 6 years after	Post-diagnostic	[45,51]
SBCSS	77-items FFQ	All food groups	6 months before BC diagnosis, after 18 months, after 36 months, and after 5 years	Mixed	[29,52,53,63]
WHEL	24 h dietary recall for 3 days (weekday 2, weekend 1)	Food and beverages consumed in the past 24 h	At enrollment	Pre-diagnostic	[54,55]
WHI	122-items WHI FFQ	All food groups	At the enrollment and each year till the 9th year	Mixed	[56,57]

Abbreviations: BC, breast cancer; BCFR, Breast Cancer Family Registry; CPS-II, Cancer Prevention Study II; CWLS, Collaborative Women’s Longevity Study; DCH, Diet, Cancer and Health; EPIC, European Prospective Investigation into Cancer and Nutrition; FFQ, food frequency questionnaire; HEAL, Health, Eating, Activity and Lifestyle Study; LACE, Life After Cancer Epidemiology Study; NHANES III, Third National Health and Nutrition Examination Survey; NHS, Nurses’ Health Study; SBCSS, Shanghai Breast Cancer Survival Study; WHEL, Women’s Healthy Eating and Living Study; WHI, Women’s Health Initiative.

Detailed characteristics of the prospective cohort studies are reported in Table 4. In several prospective cohort studies, information on tumor molecular subtypes was not reported [31,33,44,49,60]. Among studies that reported molecular characteristics, molecular subtypes included ER [32,45,47], ER/PR [29,38,39,40,41,46,48,51,52,56,58,59], ER/PR/HER2 [34,35,53], and HER2 and ER positivity [50], consistent with those assessed in RCTs.

Medical treatments varied across studies and included combinations of RT, CT, HT, and immunotherapy (IT) [29,52,53]; RT, CT, and HT [33,41,42,46,47,48,50,51]; CT and RT [38,39,40,54,59]; and CT with HT [45]. Seven studies did not report treatment information [27,28,29,30,33,43,45,46,51,52]. Cancer stage at diagnosis ranged from stage I to II [37], stage I to III [31,32,34,35,36,44,49,56,57,60], stage I to IV with an unspecified stage [34,35,52,53,58], stage I to III, with an unspecified stage [56,57], and stage I to IV [29,36,48,60]. In five studies, the stage was not reported [32,42,44,45,49]. Median follow-up ranged from 1 to 30 years, and outcomes included ACM, CSM, recurrence, OS, and DFS.

In all these studies, dietary intake was assessed using FFQs, while only studies based on the EPIC cohort also employed 7-day dietary records [34,35]. The findings from prospective cohort studies regarding the association between nutrition and BC outcomes are summarized in Table 4. These studies primarily investigated macronutrients, specific foods and beverages, dietary patterns, and adherence to predefined dietary recommendations, assessed through a variety of nutritional indices.

**Table 4:** Characteristics and main results of prospective cohort studies.

First Author, Year	Cohort Name (Country)	Tumor Receptor Status	Therapy	Years of Diagnosis	N Cases	Cancer Stage at Diagnosis	Follow-Up (Median, Years)	Type of Diet/Food Evaluated/Dietary Index	Outcomes	Main Finding
Andersen, 2020 [32]	DCH (Denmark)	ER	NR	1993–1997	977	NR	18	WG Dairy products	ACM CSM R	No significant association was observed.
Anyene, 2021 [36]	Pathways Study (USA)	ER HER2	NR	2005–2013	3646	I II III IV	9.2	PDI uPDI hPDI	R ACM	No significant association was observed.
Beasley, 2011 [33]	CWLS (USA)	NR	RT CT HT	1987–1999	4441	I II III	5.5	SFA MUFA PUFA *trans*-FA Carbohydrates Proteins	ACM CSM	Highest versus lowest quintile of SFA and *trans*-FA intake was associated with increased ACM. (HR = 1.41, 95% CI = 1.06–1.87 and HR = 1.78, 95% CI = 1.35–2.32, respectively)
Belle, 2011 [31]	HEAL (USA)	NR	NR	1996–2004	944	I II IIIA	6.7	Carbohydrates GI GL Fiber	ACM CSM R	Highest versus lowest GI intake was associated with increased R (CI not reported).
Castro-Espin, 2023 [35]	EPIC (Denmark, France, Germany, Italy, Netherlands, Norway, Spain, Sweden, UK)	ER PR HER2	NR	1992–2000	13,270	0/I II III IV Unk	8.6	DRRD ISD ERDP	ACM CSM	Higher adherence to DRRD was associated with reduced ACM. (HR = 0.92, 95% CI = 0.87–0.96).
Castro-Espin, 2023 [34]	EPIC (Denmark, France, Germany, Italy, Netherlands, Norway, Spain, Sweden, UK)	ER PR HER2	NR	1992–2000	13,270	I II III IV Unk	8.6	arMED	ACM CSM	Low vs medium arMED was associated with higher ACM. (HR = 1.13,95% CI = 1.01–1.26).In postmenopausal women, a 3-unit increase in arMED was associated with an 8% decrease in ACM.
Di Maso, 2020 [58]	NR (Italy)	ER PR	NR	1991–1994	1453	I II III-IV Unk	15	MDS	OS CSM ACM	Higher MDS was associated with improved OS (63.1% vs 53.6%).
Ernest, 2022 [54]	WHEL (USA)	ER PR	CT RT	1995–2000	3081	I II IIIA	1 to 11.2	WG Refined grain	ACM CSM	Higher intake of refined grains was associated with increased ACM (HR = 1.74, 95% CI = 1.17–2.59) and CSM (HR = 1.16, 95% CI = 1.08–1.26).
Farvid, 2021 [41]	NHS NHSII (USA)	ER PR	RT CT HT	(1980–2010) and NHSII (1991–2011)	8932	I II III	11.5	GI GL II IL	CSM ACM	Higher GI (HR = 1.23, 95% CI = 1.08–1.40), II (HR = 1.20, 95% CI = 1.04–1.38), and IL (HR = 1.23, 95% CI = 1.07–1.42) were associated with increased ACM.
Farvid, 2021 [38]	NHS NHSII (USA)	ER PR	CT RT	1976 (NHS) 1989 (NHSII)	8932	I II III	11.5	TS AS NS WGC CF CP CFJ	ACM CSM	TS is associated with an increase in ACM (HR = 1.23, 95% CI = 1.08–1.41). AS is associated with ACM (HR = 1.20, 95% CI = 1.06–1.36). CP is associated with an increase in CSM (HR = 1.25, 95% CI = 1.02–1.53).
Farvid, 2021 [39]	NHS NHSII (USA)	ER PR	CT RT	1976 (NHS) 1989 (NHSII)	8900	I II III	30	Coffee Tea	ACM CSM	Higher coffee consumption (>3 cups/day) was associated with reduced ACM (HR = 0.76, 95% CI = 0.66–0.87) and CSM (HR = 0.75, 95% CI = 0.5–0.96), while higher tea consumption (>3 cups/day) was associated with reduced ACM only (HR = 0.74, 95% CI = 0.58–0.95).
Farvid, 2021 [40]	NHS NHSII (USA)	ER PR	CT RT	1976 (NHS) 1989 (NHSII)	8863	I II III	11.5	SSBASB	ACM CSM	Consumption of SSB was associated with increased ACM (HR = 1.28, 95% CI = 1.113–1.45) and CSM (HR = 1.35, 95% CI = 1.12–1.62), whereas no association was observed with artificially sweetened beverages.
George, 2014 [57]	WHI-OS WHI-DM (USA)	ER PR	NR	1993–1998	2317	I II III Unk	9.6	HEI-2005	ACM CSM	Higher HEI-2005 score was associated with reduced ACM (HR = 0.74, 95% CI = 0.55–0.99).
Haslam, 2023 [48]	BCFR (USA, Canada, Australia)	ER PR	RT CT HT	1993–2011	6157	I II III IV	11.3	HEI-2015 AHEI aMED DASH	ACM	Higher scores of, AHEI, aMED, and DASH were associated with reduced ACM (HR = 0.82, 95% CI = 0.69–0.97), HR = 0.73, 95% CI = 0.59–0.92, and HR 0.78, 95% CI = 0.65–0.94, respectively)
Holmes, 1998 [44]	NHS (USA)	NR	NR	1976–1990	1982	NR	18	Fat Protein Red meat Fiber Poultry Dairy 80 Food items	ACM	No significant association was observed
Holmes, 2017 [47]	NHS (USA)	ER	RT CT HT	1976–2004	6348	I II III	NR	Total protein Plant protein Animal protein Red meat Poultry Fish High-fat dairy Low-fat dairy	CSM R	Intakes of total protein were associated with a decrease in R (risk reduction 4.10% at 10 years). Intakes of total protein and poultry with skin were associated with a decrease in CSM (HR = 0.79, 95% CI = 0.65–0.95) and HR = 0.76. 95% CI = 0.62–0.93, respectively).
Izano, 2013 [43]	NHS (USA)	ER	CT RT HT	1980–2003	4103	I II III	9.3	AHEI-2010 DASH	R CSM	No significant association was observed.
Jang, 2018 [59]	NR (South Korea)	ER PR HER2	CT RT	2000–2017	511	I II III	17.75	DII	DFS OS R ACM	Higher DII score was associated with reduced DFS and OS, and increased R (HR = 2.347, 95% CI = 1.17–4.71 and ACM (HR = 3.049, 95% CI = 1.08–8.83).
Karavasiloglou, 2019 [49]	NHANES III (USA)	NR	NR	1988–1994	230	NR	16	HEI MDS	ACM	Higher HEI score was associated with lower ACM (HR = 0.97, 95% CI = 0.95–0.98).
Kim, 2011 [46]	NHS (USA)	ER PR	RT CT HT	1978–1998	2729	I II III	NR	AHEI DQIR RFS aMED	ACM CSM R	No significant association was observed.
Kroenke, 2005 [45]	NHS (USA)	ER	CT HT	1982–1998	2619	NR	20	WD PD	ACM CSM	Higher adherence to a WD was associated with increased ACM and CSM
Kroenke, 2013 [50]	LACE (USA)	ER HER2	RT CT HT	1997–2000	1893	I II IIIA	11.8	Total dairy High-fat dairy Low- fat dairy	ACM CSM R	Higher intake of high-fat dairy was associated with increased ACM and CSM (HR = 1.49, 95% CI = 1.00–2.24).
Kwan, 2009 [51]	LACE (USA)	ER PR	RT CT HT	1997–2000	1901	I IIA IIB IIIA	5.9	PD WD	ACM CSM R	Higher adherence to a PD was associated with reduced ACM (HR = 0.57, 95% CI 0.36–0.90).
Mantzorou, 2022 [60]	NR (Greece)	NR	NR	2017–2018	114	I II III + IV	3.5	MDS	R	Higher adherence to the MDS was associated with reduced R
McCullough, 2016 [37]	CPS-II Nutrition Cohort (USA)	ER PR	CT RT HT	1992–2011	4452	I II	9.8	ACS guideline diet score	ACM CSM	No significant association was observed.
Shu, 2009 [29]	SBCSS (China)	ER PR	RT CT IT HT	2002–2006	5042	I II III IV	9	Soy protein	ACM R CSM	Higher soy protein intake was associated with reduced ACM (HR = 0.67, 95% CI = 0.51–0.88) and R (HR = 0.66, 95% CI = 0.52–0.84).
Sun, 2018 [56]	WHI (USA)	ER PR	NR	1993–1998	2295	I II III Unk	12	HEI-2010	ACM CSM	Lower HEI-2010 score was associated with increased CSM among postmenopausal women (HR = 1.66, 95% CI = 1.09–2.52).
Thomson, 2011 [55]	WHEL (USA)	ER	CT RT HT	1995–2006	3080	I IIA IIB IIIA IIIC	7.3	Vegetable	R	Higher vegetable intake was associated with reduced R (HR = 0.69, 95% CI = 0.55–0.87).
Wang, 2020 [53]	SBCSS (China)	ER PR HER2	RT CT HT IT	2002–2006	3450	I II III/IV Unk	5	CHFP-2007 CHFP-2016 HEI DASH	ACM CSM	Higher adherence to CHFP-2007, CHFP-2016, and DASH was associated with reduced ACM (HR = 0.66, 95% CI = 0.48–0.89; HR = 0.75, 95% CI = 0.55–1.01 and HR = 0.66, 95% CI = 0.49–0.91, respectively) and CSM (HR = 0.64, 95% CI = 0.44–0.93; HR = 0.67, 95% CI = 0.45–0.99, and HR = 0.60, 95% CI = 0.40–0.90, respectively).
Wang, 2021 [42]	NHS NHSII (USA)	ER	CT RT HT	1980–2010 NHS 1991–2015 NHSII	8482	NR	14	DRRD	CSM ACM	Higher DRRD score was associated with reduced CSM (HR = 0.77, 95% CI = 0.62–0.95) and ACM (HR = 0.85, 95% CI = 0.74–0.97).
Wang, 2022 [52]	SBCSS (China)	ER PR	RT CT HT IT	2002–2006	3575	I II III/IV Unk	8.27	Nut	OS DFS	Higher nut intake was associated with improved OS (HR = 0.72, 95% CI = 0.52–1.05) and DFS (HR = 0.48, 95% CI = 0.31–0.73) in a dose–response manner.

Higher vs. lower categories refer to study-specific quantiles as defined in the original publications. Abbreviations: ACM, all-cause mortality; ACS, American Cancer Society; AHEI, Alternate Healthy Eating Index; aMED, Alternative Mediterranean Diet; arMed, Adapted Relative Mediterranean Diet; AS, added sugars; ASB, artificially sweetened beverages; BCFR, Breast Cancer Family Registry; CF, carbohydrates from fruits; CFJ, carbohydrates from fruit juices; CHFP, Chinese Food Pagoda; CP, carbohydrates from potatoes; CPS-II, Cancer Prevention Study II; CSM, breast cancer-specific mortality; CT, chemotherapy; CWLS, Collaborative Women’s Longevity Study; DASH, Dietary Approaches to Stop Hypertension; DCH, Diet, Cancer and Health; DFS, disease-free survival; DII, dietary inflammatory index; DQIR, Diet Quality Index–Revised; DRRD, Diabetes Risk Reduction Diet; EPIC, European Prospective Investigation into Cancer and Nutrition; ER, estrogen receptor; ERDP, estrogen-related dietary pattern; GI, glycemic index; GL, glycemic load; HEAL, Health, Eating, Activity and Lifestyle Study; HEI, Healthy Eating Index; HER2, human epidermal growth factor receptor 2; hPDI, healthy plant-based index; HT, hormone therapy; II, insulin index; IL, insulin load; ISD, inflammatory score of diet; LACE, Life After Cancer Epidemiology Study; MDS, Mediterranean diet score; MUFAs, monounsaturated fatty acids; NHANES III, Third National Health and Nutrition Examination Survey; NHS, Nurses’ Health Study; NR, not reported; NS, natural sugars; OS, overall survival; PD, prudent diet; PDI, plant-based index; PR, progesterone receptor; PUFAs, polyunsaturated fatty acids; R, recurrence; RFS, Recommended Food Score; RT, radiotherapy; SBCSS, Shanghai Breast Cancer Survival Study; SFAs, saturated fatty acids; SSB, sugar-sweetened beverages; TS, total sugars; uPDI, unhealthy plant-based index; Unk, unknown; WD, Western diet; WG, whole grain; WGC, whole grain carbohydrates; WHEL, Women’s Healthy Eating and Living Study; WHI, Women’s Health Initiative.

#### 3.3.1. Macronutrients

An estimated 40–80% of cancer patients experience malnutrition during their illness, particularly protein malnutrition, which can affect treatment outcomes and survival [14,64]. Nevertheless, this important nutritional issue has not been considered in many of the prospective cohort studies.

Only three studies investigated the association between protein intake and BC outcomes. Two of these studies were conducted by Holmes et al. using the NHS cohort [44,47], and one was a prospective cohort study by Beasley et al. utilizing the CWLS cohort [33] (see Table 4). The first study by Holmes et al. reported no significant associations between low-fat or high-fat dairy consumption, red meat intake, and ACM; however, an increased survival rate was observed among women consuming more protein [44]. Beasley et al. investigated the effects of all macronutrients (protein, fat, and carbohydrate) on BC outcomes, specifically looking at the differences between various types of fatty acids (FAs), including saturated fatty acids (SFAs), monounsaturated fatty acids (MUFAs), polyunsaturated fatty acids (PUFAs), and *trans* FAs [33]. This study examined the impact of macronutrient intake on mortality in the CWLS cohort (Table 4). The results indicated no significant association between energy and macronutrient intake and ACM or CSM. The authors also explored the effects of meat and dairy products—major sources of SFA intake—on ACM and CSM. According to the results of Beasley et al. [33], there was no significant association between the consumption of meat and dairy products and the survival rates for either ACM or CSM.

In the second and most recent study, Holmes et al. found that higher protein intake was associated with a reduced risk of recurrence [47]. This association was particularly significant for protein sourced from animal products (Table 4). Additionally, higher animal protein intake was associated with a lower risk of CSM [47]. The combined findings of these studies support the results of RCTs regarding dietary fats. They suggest that neither the amount nor the type of dietary fats significantly impacts BC outcomes. Furthermore, the studies emphasize the significance of obtaining adequate protein from both animal and plant sources to prevent protein malnutrition and decrease the recurrence of BC.

Belle et al. investigated the effects of carbohydrate and fiber intake, including the glycemic index (GI) and the glycemic load (GL), on ACM, CSM, and recurrence in the HEAL cohort. No effects on the parameters investigated or on the BC outcome were found, except for an increase in recurrence in subjects on a high-GI diet compared with a low-GI diet [31] (Table 4).

Lastly, Farvid et al. explored the associations between the intake of simple carbohydrates (sugars) after BC diagnosis and CSM and ACM in the NHS and NHS II cohorts. After adjustment for confounding variables, greater post-diagnostic total sugar intake was associated with a greater risk of CSM and ACM. Higher post-diagnostic intake of added sugar (natural sugars and artificial sweeteners) was significantly associated with an increased risk of ACM. In contrast, post-diagnostic natural sugar intake was not associated with mortality risk [38] (Table 4).

#### 3.3.2. Dairy Products

Another study investigated the influence of dairy product intake on BC outcomes among the LACE cohort [50] (Table 4). After a median follow-up of 11.8 years, multivariable-adjusted analyses found no association between low-fat dairy intake and BC recurrence or survival. These results agree with the findings of Beasley with the CWLS cohort [33] and Holmes et al. with the NHS cohort [44]. In contrast with these previous studies, the authors found that high-fat dairy intake (≥1.0 serving/day) was associated with a higher risk of ACM and death from non-BC causes [50].

#### 3.3.3. Vegetables, Soy, Nuts, and Grains

Vegetable, soy, nuts, and grain intake were examined across five studies [29,32,52,54,55]. Thomson et al. investigated the association between total vegetable intake and recurrence among 3080 BC women in the WHEL study. Women in the highest tertile of vegetable intake had significantly lower recurrence, suggesting a protective effect of high vegetable intake [55].

A Danish study examined the associations between the intake of whole grains (WG) and dairy products among the DCH cohort [32]. In this cohort, there was no association between pre- or post-diagnostic intake of dairy products and BC prognosis (Table 4). The same was true for WG. However, a generally high cheese intake was associated with a higher recurrence, whereas post-diagnostic rye bread intake was significantly associated with a higher CSM. In addition, Ernest et al. found that an increase in refined grain intake compared to WG led to a rise in ACM and CSM in the US WHEL cohort [54]. For instance, for the 4th quartile of refined grains, the ACM hazard ratio (HR) was 1.74, and the CSM HR was 1.16; no protective effects of WG were observed (Table 4).

Regarding soy food intake, Shu et al. examined its effects in the SBCSS cohort of 5042 Chinese BC survivors [29]. After a median follow-up time of 3.9 years, soy food consumption, measured as soy protein intake, was inversely associated with ACM and recurrence (Table 4). The HRs comparing the highest to lowest quartiles of soy protein intake were 0.67 for ACM and 0.66 for recurrence. When soy isoflavone intake was considered, the HRs were 0.76 for ACM and 0.74 for recurrence. Interestingly, the associations of soy protein/isoflavone intake with ACM and recurrence appear to follow a linear dose–response pattern until soy protein intake reaches 11 g/day (or soy isoflavone intake, 40 mg/day). Using the same SBCSS cohort, Wang et al. examined the impact of nut consumption among 3449 long-term BC survivors [52]. At 10 years post-diagnosis, regular nut consumers had higher OS and DFS rates. After multivariable adjustment, nut consumption was positively associated with OS and DFS in a dose–response pattern, with hazard ratios of 0.72 for OS and 0.48 for DFS for participants with greater-than-median nut intake compared with no consumers (Table 4).

#### 3.3.4. Beverages

Farvid et al. examined the relationships between the consumption of nervine and sweetened beverages and BC outcomes in cohorts of women from the NHS and NHS II [39,40]. In their investigation of caffeinated beverages, they evaluated coffee and tea consumption, including decaffeinated options. The findings revealed that consuming more than three cups of coffee daily was associated with a 46% reduction in CSM and a 42% reduction in ACM. Regular coffee, which contains caffeine, was linked to a 43% reduction in CSM and a 32% reduction in ACM. Additionally, drinking three or more cups of tea daily was associated with a 29% reduction in ACM, although no effects on CSM were observed [39]. Furthermore, Farvid et al. prospectively evaluated the relationship between post-diagnostic consumption of sugar-sweetened beverages (SSBs) and artificially sweetened beverages (ASBs) and mortality rates. After a median follow-up period of 11.5 years, the study found that women who consumed SSBs after their cancer diagnosis faced a higher risk of both CSM and ACM. Specifically, women consuming 1 to 3 servings per week had a CSM HR of 1.31. In addition, those consuming more than 3 servings per week had a CSM HR of 1.35. For ACM, women consuming 1 to 3 servings per week had an HR of 1.21, and those consuming more than 3 servings per week had an HR of 1.28. In contrast, the consumption of ASBs did not show any association with an increased risk of CSM or ACM [40] (Table 4).

#### 3.3.5. Diets, Dietary Patterns, and Diet Quality Indexes

The diets, dietary patterns, and diet quality indexes considered were the Western Diet (WD), Prudent Diet (PD), Mediterranean Diet (MD), Dietary Approaches to Stop Hypertension (DASH), Diabetes Risk Reduction Diet (DRRD), Inflammatory Score of Diet (ISD), Dietary Inflammatory Index (DII), Estrogen-Related Dietary Pattern (ERDP), Healthy Eating Index (HEI), Alternate Healthy Eating Index (AHEI), dietary Glycemic Index (GI), the Mediterranean Diet Score (MDS), alternate Mediterranean Diet Score (aMED), adapted relative Mediterranean Diet Score (arMED), Diet Quality Index-Revised (DQIR), and Recommended Food Score (RFS).

The evaluation of adherence to healthy diets was conducted using several continuously updated scoring systems, including the HEI, AHEI, DRRD, ISD, DII, ERDP, DQIR, and RFS [65].

Two large cohorts, the NHS and the LACE, have been used to investigate the effects of WD and PD on ACM, CSM, and recurrence. Using the NHS cohort, Kroenke et al. investigated the relationships between the two diets and both ACM and CSM. High adherence to WD (quintile 5 vs quintile 1) was directly associated with ACM without affecting CSM. However, high PD adherence did not affect BC outcomes [45]. In contrast, Kwan et al., using the LACE cohort, found a significant association between higher adherence to PD and lower risk of ACM [45,51] (Table 4). According to the results of Kroenke et al., an increased ACM was noted for high adherence to the WD. In addition, no association was identified between recurrence and CSM with the two dietary patterns.

The broad definition of the MD complicates its identification and quantification, leading to various scoring systems. The MD’s impact on BC outcomes was investigated by using FFQs in six studies [46,48,49,58,60], while only one study corroborated the information obtained with FFQs with a 7-day dietary record [34]. Karavasiloglou et al. failed to find significant associations between MD and ACM, analyzing data from NHANES III (Table 4). The study investigated diet quality indices, specifically the HEI and the MDS, in 230 women with a diagnosis of BC or gynecological cancer. After a median follow-up of 16 years, an association between higher HEI score and lower mortality was found, but the association for MDS failed to reach statistical significance [49]. Another study evaluated MD adherence (assessed via MDS) in 114 women diagnosed with BC. After 42 months of follow-up, high MDS was independently associated with a lower recurrence [60]. In a larger Italian cohort study (*n* = 1453), adherence to MD was assessed by MDS by Di Maso et al. [58]. The high vs the low MDS led to OS of 63.1% vs 53.6%, respectively. In addition, high MDS reduced ACM without affecting CSM. Interestingly, in subjects aged > 55 years, high MDS decreased the ACM and CSM. The effects of MDS were also influenced by body mass index. High MDS reduced ACM in overweight and obese subjects, while no effects were observed for normal or underweight subjects [58] (Table 4). However, other studies suggest that adhering to the MD could improve BC prognosis using other diet quality indices. Higher diet quality index scores were related to better BC prognosis in a study by Haslam et al. that assessed associations of ACM with adherence to four diet quality indices, the aMED, HEI-2015, AHEI, and DASH indices, among 6157 North American women of the BCFR cohort (Table 4). Women in the highest vs. lowest quartile of the aMED, AHEI, and DASH indices had a lower risk of ACM. HR was 0.73 for aMED; 0.82 for AHEI; and 0.78 for DASH [48]. In their study, Castro-Espin et al. utilized data from the large EPIC cohort and analyzed results from both a 7-day dietary record and an FFQ. They discovered that a low arMED score, in comparison to a medium score, was linked to a 13% higher risk of ACM, with an HR of 1.13. Furthermore, a 3-unit increase in the arMED score was associated with an 8% reduced risk of OM, with an HR of 0.92. These findings remained consistent even when the analysis was limited to postmenopausal women, and the association was stronger among metastatic BC cases, showing an HR of 0.81 [34]. In contrast, Kim et al. found that aMED, AHEI, DQIR, and RFS were not associated with ACM, CSM, and recurrence in a cohort of 2729 women from the NHS with invasive stage I–III BC [46] (Table 4).

Two studies investigated HEI in the WHI cohort. The first, by George et al., determined the HEI-2005 scores in 2317 BC women [57]. The authors found that a higher HEI-2005 score was associated with a 26% lower risk of ACM but did not affect CSM (Table 4). The second study by Sun et al. [56] calculated the HEI-2010 of 2295 diagnosed postmenopausal women. After 12 years of follow-up, compared with women with relatively stable diet quality, women who had a 15% decrease in HEI-2010 score had a higher risk of CSM. Increased diet quality was not associated with risk of ACM, CSM, or death from other causes (Table 4).

The evaluation of adherence to dietary recommendations related to ACM and BC specific outcomes was conducted among 3450 BC survivors participating in the SBCSS [53]. Dietary data were collected using FFQ, with adherence scores calculated for the CHFP-2007, CHFP-2016, the modified DASH diet, and HEI-2015. Findings indicate that participants in the highest quartiles of CHFP-2007, CHFP-2016, and the DASH diet experienced a 25–34% reduction in the risk of ACM compared to those in the lowest quartiles (Table 4). The HR for these groups was as follows: 0.66 for CHFP-2007, 0.75 for CHFP-2016, and 0.66 for the DASH diet. Furthermore, there was a 36–40% decrease in the CSM among participants in the highest quartiles, with HRs of 0.64 for CHFP-2007, 0.67 for CHFP-2016, and 0.60 for the DASH diet [53]. In contrast, adherence scores for the HEI-2015 did not demonstrate a significant association with BC outcomes, aligning with the results reported by Haslam et al. [48]. However, Izano et al. did not find an association between the DASH score and CSM and recurrence [43] in 4103 women with BC in the NHS cohort. The authors obtained the same results when considering AHEI-2010 [43]. McCullough and colleagues analyzed CPS-II cohort data using an ACS guideline score to assess adherence and its impact on ACM and CSM. They found no link between high adherence to the ACS guidelines and mortality outcomes [37]. Anyene et al. studied the impact of a plant-based diet in the Pathways Study cohort using a Plant-Based Diet Index (PDI) and its unhealthy (uPDI) and healthy (hPDI) variants. After adjusting for confounding factors, none of the indices were linked to ACM or recurrence [36] (Table 4).

Several studies have examined the effects of adherence to various dietary patterns, specifically those related to antidiabetic, anti-inflammatory, and estrogen-related diets. A large cohort study evaluated adherence to the DRRD, ISD, and the ERDP among 13,270 BC patients participating in the EPIC study. Results from an average follow-up period of 8.6 years indicated that patients with higher adherence to the antidiabetic diet, as measured by the DRRD score, had a lower risk of ACM. Conversely, patients who adhered more closely to a pro-inflammatory diet, evaluated by the ISD score, experienced a 6% increase in mortality risk. No significant association was found between adherence to a diet that may influence estrogen levels, as assessed by the ERDP score, and mortality. Additionally, none of the dietary patterns were linked to CSM [35].

In a study conducted by Jang et al., a small cohort of 511 Korean women with stage I-III BC who underwent surgery was analyzed. The researchers found that a pro-inflammatory diet, evaluated using the DII, was significantly higher in patients who experienced recurrence compared to those who did not. This evaluation took place approximately 5.4 ± 5.2 months after diagnosis. Cox proportional hazards regression analysis indicated that a higher DII was positively associated with the risk of recurrence and ACM, even after adjusting for confounding factors [59].

Regarding DRRD and survival following BC, interesting associations were found in another cohort of more than 8000 women from the NHS and NHSII. Specifically, women with higher post-diagnostic DRRD scores had a 20% lower risk of CSM and a 34% lower risk of ACM (Table 4) [42].

Another study investigated the associations of post-diagnostic dietary diabetic-associated indexes GI, GL, II, and IL with CSM and ACM among 8932 individuals identified in the NHS and NHSII cohorts. Higher post-diagnostic GL was associated with a higher risk of both CSM and ACM. In addition, increased ACM was associated with higher GI, II, and IL. These associations were not modified by the insulin receptor or ER status of BC [41].

### 3.4. Overall Summary of Findings

A synthesis of the main findings across RCTs and prospective cohort studies is provided in Table 5, summarizing the direction and consistency of associations between post-diagnostic dietary exposures and BC outcomes.

Across RCTs and cohort studies, dietary intake and adherence were assessed almost exclusively using self-reported FFQs, with only a limited number of studies incorporating dietary records to complement FFQ data. Differences in exposure definition and assessment tools likely contributed to heterogeneity in observed association. Collectively, evidence from RCTs and prospective cohort studies suggests that post-diagnostic dietary habits exhibit limited and inconsistent effects on BC–specific outcomes. Conversely, adherence to healthier dietary patterns and enhanced diet quality are more consistently associated with reduced ACM. It is important to acknowledge that a significant proportion of the studies included in this analysis originated from a limited number of large cohorts, primarily located in the United States. These findings should be interpreted with caution due to the significant methodological heterogeneity observed across the studies. This heterogeneity encompasses variations in dietary assessment methodologies, definitions of exposure, and metrics of outcomes.

## 4. Discussion

This systematic review synthesizes evidence from eight randomized controlled trials and thirty-one prospective cohort studies examining the association between post-diagnostic dietary factors and BC outcomes. In addition to updating the available evidence base, this review provides a structured and methodologically informed synthesis, with particular emphasis on heterogeneity across dietary assessment methods, exposure definitions, and nutritional considerations. Importantly, RCTs and observational studies provide different levels of evidence and are subject to distinct sources of bias. While RCTs allow stronger causal inference, their findings in this field were limited and often affected by methodological constraints. In contrast, cohort studies provide valuable real-world evidence but remain susceptible to residual confounding. The interpretation of findings is further limited by the frequent use of relative comparisons (e.g., highest vs lowest intake categories), which may not reflect clinically meaningful differences in absolute intake. The decision to synthesize evidence from randomized controlled trials and prospective cohort studies within the same review was intentional and reflects the objective of providing a comprehensive evaluation of both interventional and observational evidence addressing the same clinical question. This integrated approach allows direct comparison of findings across complementary study designs while highlighting areas of consistency and uncertainty in the current evidence base.

Overall, the evidence is heterogeneous and does not support consistent associations between specific dietary components and BC-specific outcomes. Current evidence does not consistently support an association between post-diagnostic diet and breast cancer–specific outcomes. However, a more consistent pattern emerges for ACM, with healthier dietary patterns and higher diet quality scores generally associated with improved overall survival. This heterogeneity reflects multiple methodological and clinical dimensions observed across the included studies, as detailed in the Section 2.5, including variability in dietary assessment methods, timing of exposure assessment (pre- vs post-diagnosis), heterogeneity in dietary exposure definitions and categorization (e.g., nutrients, foods, patterns, and indices), differences in clinical characteristics (cancer stage, receptor status, and treatments), as well as variability in follow-up duration and outcome definitions. In addition, alcohol intake was not evaluated as an independent exposure but was variably included within dietary patterns and indices, further contributing to heterogeneity in exposure definitions across studies. These findings should be interpreted in light of the multifactorial nature of the observed heterogeneity, which reflects the combined influence of methodological and clinical variability rather than any single determinant.

In light of these findings, we also provide an overall qualitative assessment of the certainty of the evidence across the main exposure–outcome categories. Considering key domains such as study design, risk of bias, consistency of findings, and methodological limitations, the overall certainty of evidence was judged to be low–to–moderate. In particular, the certainty of evidence was low for most associations involving individual nutrients and food groups, low–to–moderate for associations between dietary patterns or diet quality indices and all-cause mortality, and low for breast cancer–specific outcomes (including breast cancer–specific mortality), reflecting heterogeneity in outcome definitions and inconsistency of findings across studies. These considerations are consistent with the methodological heterogeneity described in the Section 2.5 and further support the interpretation of the findings within a heterogeneous evidence base.

Importantly, these findings must be interpreted considering the structure and quality of the available evidence base. A substantial proportion of included studies originated from a limited number of large, well-established cohorts, predominantly based in the United States (e.g., WHI, NHS/NHSII, WHEL, LACE, Pathways). As a result, the apparent diversity in the number of studies may overestimate the true diversity of independent populations. Although this concentration may partly reflect the structure of the available literature, the possibility that some eligible studies from other geographical regions were not identified cannot be excluded. Therefore, the generalizability of the findings to populations with different dietary patterns, cultural contexts, and healthcare systems should be interpreted with caution.

As summarized in Table 5, healthier dietary patterns and higher diet quality scores were more consistently associated with reduced ACM than with BC–specific outcomes, highlighting the potential role of post-diagnostic diet in improving overall survivorship rather than directly influencing disease-specific prognosis. However, these findings are primarily derived from observational studies and should be interpreted with caution due to the potential for residual confounding.

Another key consideration is the overall methodological quality of the included studies. The risk-of-bias assessment revealed that most randomized controlled trials were at high risk of bias, while most cohort studies were of moderate quality. This evidence profile has important implications for interpretation. The predominance of studies at moderate-to-high risk of bias reduces confidence in observed associations and may contribute to the inconsistency of findings across studies. In particular, the high risk of bias identified in most randomized trials limits the strength of causal inference, despite the interventional design. At the same time, observational studies are inherently susceptible to residual confounding, especially in the context of complex exposures such as diet. Therefore, the absence of consistent associations should not be interpreted as definitive evidence of no effect, but rather as reflecting limitations in the current evidence base.

Measurement issues in dietary assessments represent a significant challenge for studies that link diet to diseases, including cancer. Although the widespread use of FFQs is likely to introduce measurement error and may contribute to variability in observed associations, no formal comparison across dietary assessment methods was conducted in this review. Therefore, dietary assessment methods should be interpreted as potential contributors to heterogeneity rather than definitive explanations for inconsistent findings. Despite extensive efforts to develop new nutritional assessment tools using modern technology, FDs are often considered a more precise method for short-term intake [10], although they are also subject to limitations, including participant burden and limited ability to capture habitual dietary patterns. In addition, the act of recording food intake may influence participants’ behavior (reactivity), further complicating interpretation [65].

A study by Bingham et al., as early as 2002, highlighted a notable difference in outcomes between FFQs and FDs. However, in the reviewed studies, patients’ dietary habits were mainly assessed using FFQs. Only two of these studies, which focused on the EPIC cohort, also utilized a 7-day FD [34,35]. The most relevant finding emerging from this review is not the identification of a single dietary exposure consistently associated with improved breast cancer outcomes, but rather the characterization of the current evidence base itself. The literature remains highly heterogeneous, relies heavily on a limited number of cohorts, and is affected by important methodological limitations. Consequently, the principal contribution of this review is the identification of these limitations and evidence gaps, which currently preclude strong conclusions and highlight priorities for future research.

Beyond issues related to dietary assessment, which have been extensively discussed in the literature, broader methodological limitations of the evidence base appear to play a central role in shaping the observed results. These include variability in study quality, limited adjustment for key confounders, and heterogeneity in populations and clinical characteristics.

The principal confounding factors that are frequently inadequately addressed include nutritional status, which encompasses both malnutrition—defined as undernutrition or overnutrition—and insufficient differentiation of older populations and menopausal status, as well as disease-related variables.

Current nutritional guidelines for cancer patients recommend maintaining a good nutritional state to prevent common complications such as weight loss and malnutrition [66,67]. However, these recommendations are often overlooked in clinical practice and not considered in studies related to nutritional issues. Even though there is currently no universally accepted gold standard (best method) for assessing nutritional status, it has traditionally been identified through various methods, including anthropometric, biochemical, and physical assessments [68]. In the studies we analyzed, malnutrition was overlooked and, when considered, was primarily evaluated using anthropometric measures such as BMI [58], which do not fully capture the multidimensional nature of malnutrition. As a result, available evidence largely reflects associations with indicators such as underweight or body mass index, rather than malnutrition as a comprehensive clinical condition. However, BMI has important limitations, particularly in cancer populations, as it may not adequately reflect true nutritional status. It does not distinguish between fat and lean mass, does not evaluate individual health, or take personal factors into account. It should be noted that BMI is often one of the most important components of screening tools for malnutrition [69]. Recent revisions have updated the definition of malnutrition and obesity based on BMI criteria to offer a more accurate and detailed understanding of obesity and its related health risks [69,70]. The new framework for defining and diagnosing obesity goes beyond BMI and now includes waist circumference, body fat, lean mass measurements, and health markers. Consequently, the role of malnutrition per se in breast cancer prognosis remains insufficiently characterized in the current evidence base.

In addition, only three of the studies analyzed consider the effects of age and menopausal status on BC outcomes [34,35,56]. However, both these conditions are associated with differences in treatment tolerance, metastatic patterns, and prognosis [71,72].

Regarding variables related to disease, there are BC’s stage, location, and type, along with the treatments administered and their associated side effects [9]. The interaction between these factors and nutritional variables makes it difficult to draw definitive conclusions from observational studies [71,72].

Finally, additional biological mechanisms, including epigenetic pathways and factors influencing appetite (e.g., supportive treatments), may play a role in shaping dietary behaviors and outcomes. However, these aspects were not systematically assessed in the included studies. Future research integrating dietary assessment with molecular and epigenetic data may help clarify these relationships. 

Within these constraints, the most consistent signal suggests that overall diet quality may contribute to general health and survival, as reflected in associations with all-cause mortality, rather than exerting a strong and direct effect on breast cancer–specific outcomes. This interpretation is consistent with the overall low–to–moderate certainty of evidence and the substantial heterogeneity observed across studies. Accordingly, observed associations should be interpreted cautiously, particularly given the observational nature of much of the evidence and the potential for residual confounding.

### Limitations and Future Directions

This systematic review has several limitations that should be acknowledged when interpreting the findings. First, substantial heterogeneity across studies was observed in terms of dietary assessment methods, study populations, cancer characteristics, and outcome definitions. The predominant use of FFQs, although widely accepted in epidemiological research, is subject to recall bias and measurement error, which may have contributed to exposure misclassification and attenuation of true associations.

Second, the included studies often lacked a comprehensive assessment of important confounding factors. Nutritional status was rarely evaluated beyond body mass index (BMI), which does not adequately reflect body composition or distinguish between fat and lean mass. The absence of standardized and multidimensional measures of malnutrition may have limited the ability to detect clinically meaningful associations between diet and BC outcomes.

Third, incomplete reporting of clinical variables, including cancer stage, molecular subtype, treatment regimens, and menopausal status, further complicates the interpretation of results. Although many studies considered some of these factors, their inconsistent inclusion and adjustment limit cross-study comparability. Additionally, potential effects of supportive and palliative treatments, such as cannabinoids, which may influence appetite and nutritional status, were not systematically accounted for.

Fourth, the observational nature of most included studies precludes causal inference. Residual confounding, particularly from lifestyle factors such as physical activity, comorbidities, and socioeconomic status, cannot be excluded. Furthermore, variability in follow-up duration and timing of dietary assessment relative to diagnosis may have influenced the observed associations.

Fifth, although the review focused on post-diagnostic diet, some included studies assessed dietary intake before diagnosis or at cohort enrollment. These measures may not capture dietary changes following diagnosis and could reflect long-term dietary habits instead. This heterogeneity in exposure timing may have contributed to variability in findings.

Finally, numerous studies have reported associations based on study-specific categories of intake or dietary scores, yet they frequently lack detailed quantitative information regarding the corresponding levels of food or nutrient consumption. This deficiency restricts the ability to assess clinically significant differences in exposure and diminishes the translational applicability of the results. This also makes it difficult to translate observed associations into practical dietary recommendations for BC survivors. Furthermore, a substantial portion of the evidence arises from a limited number of large cohorts, predominantly situated in the United States. While this may partially reflect the existing literature, it remains plausible that some pertinent international studies were overlooked by the search strategy. Therefore, it is imperative to exercise caution when generalizing these findings across diverse geographical and cultural contexts.

Future research should prioritize the use of more precise and objective dietary assessment tools, including repeated dietary measures and biomarkers of nutritional intake, to improve exposure accuracy. Standardized definitions and validated tools for assessing nutritional status, including body composition and functional measures, should be incorporated into both observational studies and clinical trials. Moreover, future studies should adopt comprehensive approaches that integrate dietary data with detailed clinical, metabolic, and molecular information, including tumor subtype and treatment characteristics.

Randomized controlled trials with well-defined dietary interventions, clear adherence monitoring, and clinically relevant endpoints are needed to clarify the potential role of nutrition in BC prognosis. Finally, emerging areas such as the interaction between diet, epigenetic mechanisms, and cancer progression warrant further investigation to better understand the biological pathways linking nutrition to BC outcomes and to support the development of personalized nutritional strategies.

## 5. Conclusions

The findings of this systematic review suggest that healthier dietary patterns and higher diet quality scores may be associated with improved survival outcomes, particularly in relation to all-cause mortality. However, these findings are largely based on observational evidence and remain susceptible to residual confounding. These results are consistent with the observation that post-diagnostic diet may play a more relevant role in overall survival and general health status rather than directly influencing disease-specific prognosis. However, these findings should be interpreted with caution due to important methodological limitations, including the widespread use of imprecise dietary assessment tools and the incomplete consideration of key confounding factors. Inadequate evaluation of nutritional status and body composition, as well as heterogeneity in clinical characteristics across studies, may have contributed to the inconclusive nature of the evidence.

The main contribution of this review lies not in generating a summary quantitative estimate, but in integrating updated evidence with a detailed methodological appraisal of the literature. By systematically examining the heterogeneity, limitations, and overall certainty of the available evidence, this review offers a more nuanced interpretation of the role of post-diagnostic diet in breast cancer survivorship and identifies key priorities for future research.

Current nutritional guidelines for cancer patients emphasize the importance of maintaining adequate nutritional status and sufficient protein intake to prevent complications such as malnutrition and weight loss. Nevertheless, these recommendations are often inconsistently implemented in clinical practice and are not systematically incorporated into nutritional research.

Importantly, the evidence base remains characterized by substantial heterogeneity in study design, dietary exposure definitions, dietary assessment methods, outcome reporting, and clinical characteristics of included populations. In addition, a considerable proportion of the available evidence originates from a limited number of cohorts, further constraining the diversity and independence of the literature.

Future studies are needed to clarify whether specific dietary patterns can influence BC outcomes and to better understand the biological mechanisms underlying these associations. Research should incorporate more accurate dietary assessment methods, such as FDs and objective biomarkers, along with comprehensive and standardized evaluation of nutritional status. Such approaches will be essential for developing evidence-based and personalized nutritional strategies to support long-term survivorship in BC patients.

Taken together, the findings suggest that the current evidence base is not yet sufficiently consistent, comparable, or methodologically robust to support strong conclusions regarding specific post-diagnostic dietary recommendations for breast cancer outcomes. The identification of these limitations and evidence gaps represents a central finding of this review and highlights the need for future high-quality studies capable of providing more definitive evidence.

Ultimately, advancing this field will require integrative and methodologically robust research capable of moving beyond associative evidence toward actionable, evidence-based nutritional strategies for improving long-term outcomes in BC survivors.

## Figures and Tables

**Table 1:** PICOS design for the formulation of eligibility criteria.

PICOS Component	Eligibility Criteria
Population	Women aged ≥18 years with BC
Intervention/Exposure	Dietary intervention (DP, food, macronutrient, and nutritional index)
Comparison	For RCTs: control group. For cohort studies: categories of dietary exposure (e.g., tertiles, quintiles, adherence scores)
Outcome	OS; ACM; CSM; CP; DFS and R
Study design	Cohort studies and RCTs

Abbreviations: ACM, All-Cause Mortality; CP, Cancer Progression; CSM, Breast Cancer-Specific Mortality; DFS, Disease-Free Survival; DP, Dietary Pattern; OS, Overall Survival; R, Recurrence.

**Table 5:** Summary of salient findings on post-diagnostic diet and breast cancer outcomes.

Dietary Exposure	Study Design/Population	Key Findings	Outcomes	References
Low-fat diet (LFD)	WINS, WHI, WHEL	Predominantly null or inconsistent effects on BC-specific outcomes; ↓ R in WINS; no consistent OS benefit	R, OS, ACM	[21,22,23,24,25,26,27,30]
Mediterranean–macrobiotic dietary intervention (DIANA-5)	DIANA-5 RCT	No significant effect on BC recurrence	R	[28]
Protein intake	Cohort studies (NHS, CWLS)	Higher intake associated with ↓ R and ↓ CSM; no consistent association with ACM	R, CSM, ACM	[33,44,47]
Carbohydrates (GI/GL, sugars)	Cohort studies (HEAL, NHS/NHSII)	High GI/GL and sugar intake associated with ↑ R, ↑ CSM, and ↑ ACM	R, CSM, ACM	[31,38,41]
Dairy products	Cohort studies (LACE, NHS, CWLS)	No association for low-fat dairy; high-fat dairy associated with ↑ ACM	ACM, CSM, R	[33,44,50]
Vegetables and grains	Cohort studies (WHEL, DCH)	Higher vegetable intake associated with ↓ R; refined grains associated with ↑ ACM and ↑ CSM	R, ACM, CSM	[32,54,55]
Soy foods and nuts	Cohort studies (SBCSS)	Soy intake associated with ↓ ACM and ↓ R; nut consumption associated with improved OS and DFS	OS, DFS, ACM, R	[29,52]
Beverages (coffee, tea, SSBs)	Cohort studies (NHS/NHSII)	Coffee associated with ↓ ACM and ↓ CSM; tea associated with ↓ ACM only; SSBs associated with ↑ ACM and ↑ CSM	ACM, CSM	[39,40]
Western vs. prudent dietary patterns	Cohort studies (NHS, LACE)	Western diet associated with ↑ ACM; prudent diet associated with ↓ ACM; no consistent association with CSM	ACM, CSM	[45,51]
Mediterranean diet and diet quality indices	Cohort studies (EPIC, NHS, BCFR, NHANES)	Mixed results; some indices associated with ↓ ACM; inconsistent findings for CSM and R	ACM, CSM, R	[34,46,48,49,58,60]
Healthy eating indices (HEI, DASH, CHFP)	Cohort studies (WHI, SBCSS, NHS)	Higher adherence associated with ↓ ACM and, in some studies, ↓ CSM; no consistent association with R	ACM, CSM	[43,53,56,57]
Plant-based diet	Cohort studies (Pathways Study)	No significant association with ACM or R	ACM, R	[36]
Inflammatory and metabolic dietary patterns (DII, DRRD, ERDP)	Cohort studies (EPIC, NHS/NHSII, Korean cohort)	Anti-diabetic patterns associated with ↓ ACM and ↓ CSM; pro-inflammatory diet associated with ↑ R and ↑ ACM	ACM, CSM, R	[35,42,59]

Abbreviations: ↑, increased; ↓, decreased; ACM, all-cause mortality; CSM, breast cancer-specific mortality; CWLS, Collaborative Women’s Longevity Study; DCH, Diet, Cancer and Health; DFS, disease-free survival; EPIC, European Prospective Investigation into Cancer and Nutrition; GI, glycemic index; GL, glycemic load; HEAL, Health, Eating, Activity and Lifestyle Study; NHS, Nurses’ Health Study; OS, overall survival; R, recurrence; RCTs, randomized controlled trials; SBCSS, Shanghai Breast Cancer Survival Study; WHI, Women’s Health Initiative; WHEL, Women’s Healthy Eating and Living Study; WINS, Women’s Intervention Nutrition Study.

## Data Availability

No new data were created or analyzed in this study. Data sharing is not applicable to this article.

## Supplementary Materials

The following supporting information can be downloaded at https://www.mdpi.com/article/10.3390/nu18162586/s1, PRISMA 2020 Checklist; Table S1. Full search strategy for PubMed/MEDLINE; Table S2. Full search strategy for Scopus; Table S3. Full search strategy for CENTRAL; Table S4. Risk-of-bias assessment of randomized controlled trials; Table S5. Risk-of-bias assessment of prospective cohort studies.

## Abbreviations

The following abbreviations are used in this manuscript:

ACM	All-Cause Mortality
ACS	American Cancer Society
AHEI	Alternate Healthy Eating Index
aMED	Alternative Mediterranean Diet
arMED	Adapted Relative Mediterranean Diet
ASB	Artificially Sweetened Beverages
BC	Breast Cancer
BCFR	Breast Cancer Family Registry
BMI	Body Mass Index
CHFP	Chinese Food Pagoda
CPS-II	Cancer Prevention Study II
CSM	Breast Cancer-Specific Mortality
CT	Chemotherapy
CWLS	Collaborative Women’s Longevity Study
DASH	Dietary Approaches to Stop Hypertension
DCH	Diet, Cancer, and Health
DFS	Disease-Free Survival
DII	Dietary Inflammatory Index
DQIR	Diet Quality Index-Revised
DRRD	Diabetes Risk Reduction Diet
EPIC	European Prospective Investigation into Cancer and Nutrition
ER	Estrogen Receptor
ERDP	Estrogen-Related Dietary Pattern
FFQ	Food Frequency Questionnaire
GI	Glycemic Index
GL	Glycemic Load
HEAL	Health, Eating, Activity, and Lifestyle
HEI	Healthy Eating Index
HER2	Human Epidermal Growth Factor Receptor 2
hPDI	Healthy Plant-Based Diet Index
HR	Hazard Ratio
HT	Hormone Therapy
II	Insulin Index
IL	Insulin Load
ISD	Inflammatory Score of Diet
LACE	Life After Cancer Epidemiology Study
MD	Mediterranean Diet
MDS	Mediterranean Diet Score
MUFAs	Monounsaturated Fatty Acids
NHANES III	Third National Health and Nutrition Examination Survey
NHS	Nurses’ Health Study
OS	Overall Survival
PD	Prudent Diet
PDI	Plant-Based Diet Index
PR	Progesterone Receptor
PUFAs	Polyunsaturated Fatty Acids
R	Recurrence
RFS	Recommended Food Score
RT	Radiotherapy
SBCSS	Shanghai Breast Cancer Survival Study
SFAs	Saturated Fatty Acids
SSB	Sugar-Sweetened Beverages
uPDI	Unhealthy Plant-Based Diet Index
WD	Western Diet
WHEL	Women’s Healthy Eating and Living
WHI	Women’s Health Initiative
