# Supplementary material for: Post-Diagnostic Diet and Breast Cancer Outcomes: A Comprehensive Systematic Review of Prospective Cohort Studies and Randomized Clinical Trials
Source: Nutrients. 2026 Aug 7;18(16):2586. doi: 10.3390/nu18162586 (PMC13516070; doi:10.3390/nu18162586)
Supplement: Supplementary file 1 [file nutrients-18-02586-s001.zip › nutrients-4389206-supplementary.pdf]

PRISMA 2020 Checklist

Section and Topic	Item #	Checklist item	Location where item is reported
**TITLE**			
Title	1	Identify the report as a systematic review.	Page 1
**ABSTRACT**			
Abstract	2	See the PRISMA 2020 for Abstracts checklist.	Page 1
**INTRODUCTION**			
Rationale	3	Describe the rationale for the review in the context of existing knowledge.	Page 1 and 2
Objectives	4	Provide an explicit statement of the objective(s) or question(s) the review addresses.	Pages 2
**METHODS**			
Eligibility criteria	5	Specify the inclusion and exclusion criteria for the review and how studies were grouped for the syntheses.	Page 3
Information sources	6	Specify all databases, registers, websites, organisations, reference lists and other sources searched or consulted to identify studies. Specify the date when each source was last searched or consulted.	Page 3
Search strategy	7	Present the full search strategies for all databases, registers and websites, including any filters and limits used.	Table A1, A2 and A3
Selection process	8	Specify the methods used to decide whether a study met the inclusion criteria of the review, including how many reviewers screened each record and each report retrieved, whether they worked independently, and if applicable, details of automation tools used in the process.	Page 4
Data collection process	9	Specify the methods used to collect data from reports, including how many reviewers collected data from each report, whether they worked independently, any processes for obtaining or confirming data from study investigators, and if applicable, details of automation tools used in the process.	Page 4
Data items	10a	List and define all outcomes for which data were sought. Specify whether all results that were compatible with each outcome domain in each study were sought (e.g. for all measures, time points, analyses), and if not, the methods used to decide which results to collect.	Pages 4
	10b	List and define all other variables for which data were sought (e.g. participant and intervention characteristics, funding sources). Describe any assumptions made about any missing or unclear information.	Table 1,2,3,4,5
Study risk of bias assessment	11	Specify the methods used to assess risk of bias in the included studies, including details of the tool(s) used, how many reviewers assessed each study and whether they worked independently, and if applicable, details of automation tools used in the process.	Page 4
Effect measures	12	Specify for each outcome the effect measure(s) (e.g. risk ratio, mean difference) used in the synthesis or presentation of results.	Tables 1, 2, and 4
Synthesis methods	13a	Describe the processes used to decide which studies were eligible for each synthesis (e.g. tabulating the study intervention characteristics and comparing against the planned groups for each synthesis (item #5)).	Tables 2; 3; 4 and 5
	13b	Describe any methods required to prepare the data for presentation or synthesis, such as handling of missing summary statistics, or data conversions.	Page 4
	13c	Describe any methods used to tabulate or visually display results of individual studies and syntheses.	Tables 2; 3; 4 and 5
	13d	Describe any methods used to synthesize results and provide a rationale for the choice(s). If meta-analysis was performed, describe the model(s), method(s) to identify the presence and extent of statistical heterogeneity, and software package(s) used.	Data Synthesis (lines 252–287) Tables 2-5
	13e	Describe any methods used to explore possible causes of heterogeneity among study results (e.g. subgroup analysis, meta-regression).	none
	13f	Describe any sensitivity analyses conducted to assess robustness of the synthesized results.	none

Section and Topic	Item #	Checklist item	Location where item is reported
Reporting bias assessment	14	Describe any methods used to assess risk of bias due to missing results in a synthesis (arising from reporting biases).	Tables A4 and A5
Certainty assessment	15	Describe any methods used to assess certainty (or confidence) in the body of evidence for an outcome.	Tables 2; 3; 4 and 5
**RESULTS**			
Study selection	16a	Describe the results of the search and selection process, from the number of records identified in the search to the number of studies included in the review, ideally using a flow diagram.	Pages 4-6
	16b	Cite studies that might appear to meet the inclusion criteria, but which were excluded, and explain why they were excluded.	Pages 4-6
Study characteristics	17	Cite each included study and present its characteristics.	Pages 6-10, tables 2 and 4
Risk of bias in studies	18	Present assessments of risk of bias for each included study.	Tables A4 and A5
Results of individual studies	19	For all outcomes, present, for each study: (a) summary statistics for each group (where appropriate) and (b) an effect estimate and its precision (e.g. confidence/credible interval), ideally using structured tables or plots.	Tables 2 and 4
Results of syntheses	20a	For each synthesis, briefly summarise the characteristics and risk of bias among contributing studies.	Pages 5-6
	20b	Present results of all statistical syntheses conducted. If meta-analysis was done, present for each the summary estimate and its precision (e.g. confidence/credible interval) and measures of statistical heterogeneity. If comparing groups, describe the direction of the effect.	none
	20c	Present results of all investigations of possible causes of heterogeneity among study results.	none
	20d	Present results of all sensitivity analyses conducted to assess the robustness of the synthesized results.	none
Reporting biases	21	Present assessments of risk of bias due to missing results (arising from reporting biases) for each synthesis assessed.	none
Certainty of evidence	22	Present assessments of certainty (or confidence) in the body of evidence for each outcome assessed.	Discussion
**DISCUSSION**			
Discussion	23a	Provide a general interpretation of the results in the context of other evidence.	Pages 22-25
	23b	Discuss any limitations of the evidence included in the review.	Pages 22-25
	23c	Discuss any limitations of the review processes used.	Pages 22-25
	23d	Discuss implications of the results for practice, policy, and future research.	Pages 22-25
**OTHER INFORMATION**			
Registration and protocol	24a	Provide registration information for the review, including register name and registration number, or state that the review was not registered.	Page 4
	24b	Indicate where the review protocol can be accessed, or state that a protocol was not prepared.	Prospero
	24c	Describe and explain any amendments to information provided at registration or in the protocol.	none
Support	25	Describe sources of financial or non-financial support for the review, and the role of the funders or sponsors in the review.	Page 26
Competing interests	26	Declare any competing interests of review authors.	Page 26
Availability of data, code and other materials	27	Report which of the following are publicly available and where they can be found: template data collection forms; data extracted from included studies; data used for all analyses; analytic code; any other materials used in the review.	Supplementary, appendix

From: Page MJ, McKenzie JE, Bossuyt PM, Boutron I, Hoffmann TC, Mulrow CD, et al. The PRISMA 2020 statement: an updated guideline for reporting systematic reviews. BMJ 2021;372:n71. doi: 10.1136/bmj.n71. This work is licensed under CC BY 4.0. To view a

copy of this license, visit https://creativecommons.org/licenses/by/4.0/

Table S1. Updated Search Strategy: PubMed-MEDLINE, 5 May 2026 (initial search: 27 May 2025).

SET	Search Terms	Search Type	Results
1	diet OR dietary OR dietary pattern OR food OR foods OR beverage OR beverages OR bread OR cereals OR grains OR whole-grain OR soy OR soya OR potatoes OR legumes OR pulses OR rice OR pasta OR vegetables OR fruit OR milk OR dairy OR eggs OR meat OR fish OR seafood OR nuts OR sweets OR alcohol OR coffee OR tea OR juice OR dietary fats OR "fatty acids" OR dietary carbohydrates OR dietary protein OR fibre OR fiber OR sugar	Advanced	5,329,866
2	Breast cancer OR Breast tumor	advanced	520,519
3	Patients OR patient	advanced	8,923,330
4	1 AND 2 AND 3	advanced	12,534
5	4 NOT risk NOT parenteral NOT enteral NOT vitamin NOT mineral NOT supplement	advanced	8,212
6	Refined by clinical trials AND Observational studies AND English language AND Humans	advanced	1,100

Note: Search terms were developed iteratively to maximize sensitivity and specificity. PubMed automatic term mapping was used to identify indexed terms and spelling variants.

Table S2. Updated Search Strategy: SCOPUS, 5 May 2026 (initial search: 27 May 2025).

SET	Search Terms	Search Type	Results
1	diet OR dietary OR dietary pattern OR food OR foods OR beverage OR beverages OR bread OR cereals OR grains OR whole-grain OR soy OR soya OR potatoes OR legumes OR pulses OR rice OR pasta OR vegetables OR fruit OR milk OR dairy OR eggs OR meat OR fish OR seafood OR nuts OR sweets OR alcohol OR coffee OR tea OR juice OR dietary fats OR "fatty acids" OR dietary carbohydrates OR dietary protein OR fibre OR fiber OR sugar	Advanced	1,211,431
2	Breast cancer OR Breast tumor	advanced	1,700,409
3	Patients OR patient	advanced	17,167,472
4	1 AND 2 AND 3	advanced	66,306
5	Refined by clinical trials AND Observational studies AND English language AND Humans	advanced	2,305

Table S3. Updated Search Strategy: CENTRAL Cochrane, 5 May 2026 (initial search: 27 May 2025).

SET	Search Terms	Search Type	Results

1	diet OR dietary OR dietary pattern OR food OR foods OR beverage OR beverages OR bread OR cereals OR grains OR whole-grain OR soy OR soya OR potatoes OR legumes OR pulses OR rice OR pasta OR vegetables OR fruit OR milk OR dairy OR eggs OR meat OR fish OR seafood OR nuts OR sweets OR alcohol OR coffee OR tea OR juice OR dietary fats OR "fatty acids" OR dietary carbohydrates OR dietary protein OR fibre OR fiber OR sugar	Advanced	271,539
2	Breast cancer OR Breast tumor	advanced	20,200
3	Patients OR patient	advanced	236,531
4	1 AND 2 AND 3	advanced	2,642

Table S4. Risk of Bias Assessment. Cochrane RoB 2 Tool of the RCTs.

	**Risk of bias domains**					
**Study**	**D1** *Randomization*	**D2** *Deviations*	**D3** *Missing data*	**D4** *Measurement*	**D5** *Reported results*	**Overall**
Berrino et al. 2024 [28]	+	X	X	+	+	**High risk**
Chlebowski et al. 2006 [22]	-	X	+	-	+	**High risk**
Chlebowski et al. 2017 [23]	X	X	-	+	+	**High risk**
Chlebowski et al. 2018 [24]	X	X	-	+	+	**High risk**
Chlebowski et al. 2020 [25]	X	X	-	+	+	**High risk**
Pierce et al 2008 [27]	+	-	+	-	+	**Some concerns**
Reddy et al 2005 [21]	-	X	+	-	+	**High risk**
Zheng et al 2018 [26]	X	X	-	+	+	**High risk**

Domains: D1 = Bias arising from the randomization process; D2 = Bias due to deviations from intended interventions; D3 = Bias due to missing outcome data; D4 = Bias in measurement of the outcome; D5 = Bias in selection of the reported result. Judgment: +, Low risk; −, Some concerns, and X, High risk.

Table S5. Newcastle-Ottawa Scale (NOS) table of prospective studies

**Study**	**Selection**	**Comparability**	**Outcome**	**Overall star rating**
Andersen et al. 2020 [32]	★★★	★★	★★	7
Anyene et al. 2021 [36]	★★★	★★	★★	7
Beasley et al. 2011 [33]	★★★	★★	★★	7
Belle et al. 2011 [31]	★★★	★★	★	6
Castro-Espin et al 2023 [35]	★★★	★★	★	6
Castro-Espin et al 2023 [34]	★★★	★★	★	6

Di Maso et al. 2020 [58]	★★★	★	★	5
Ernest et al. 2022 [54]	★★★★	★	★	6
Farvid et al. 2021 [41]	★★★	★★	★★	7
Farvid et al. 2021 [38]	★★★	★★	★★	7
Farvid et al. 2021 [39]	★★★	★★	★★	7
Farvid et al. 2020 [40]	★★★	★★	★★	7
George et al. 2014 [57]	★★★	★	★★★	7
Haslam et al. 2023 [48]	★★★★	★★	★★★	9
Holmes et al. 1998 [44]	★★★	★	★	5
Holmes et al. 2017 [47]	★★★	★★	★★	7
Izano et al. 2013 [43]	★★★	★★	★	6
Jang et al. 2018 [59]	★★	★	★★	5
Karavasiloglou et al. 2019 [49]	★★★	★★	★★★	8
Kim et al. 2011 [46]	★★★	★★	-	5
Kroenke et al. 2005 [45]	★★★	★★	★★	7
Kroenke et al. 2013 [50]	★★★	★★	★★	7
Kwan et al. 2009 [51]	★★★	★★	★	6
Mantzorou et al. 2022 [60]	★★	★	-	3
McCullough et al 2016 [37]	★★★	★★	★★	7
Shu et al. 2009 [29]	★★★	★★	★★★	8
Sun et al. 2018 [56]	★★★	★★	★	6
Thomson et al 2011 [55]	★★★★	★★	★	7
Wang et al. 2020 [53]	★★★	★★	★★	7
Wang et al. 2021 [42]	★★★★	★★	★★	8
Wang et al. 2021 [52]	★★★	★★	★	6

Maximum score: 9 stars (★), Low risk ≥ 8, Moderate 6–7, High risk ≤ 5.
